# Combining Contamination Indices and Multivariate Statistical Analysis for Metal Pollution Evaluation during the Last Century in Lacustrine Sediments of Lacu Sărat Lake, Romania

**DOI:** 10.3390/ijerph20021342

**Published:** 2023-01-11

**Authors:** Iolanda-Veronica Ganea, Ramona Bălc, Robert-Csaba Begy, Ioan Tanțău, Delia Maria Gligor

**Affiliations:** 1 Faculty of Environmental Science and Engineering, Babeș-Bolyai University, 30 Fântânele, 400294 Cluj-Napoca, Romania; 2 National Institute for Research and Development of Isotopic and Molecular Technologies, 67-103 Donath, 400293 Cluj-Napoca, Romania; 3 Interdisciplinary Research Institute on Bio-Nano-Sciences, Babeş-Bolyai University, 42 Treboniu Laurian, 400271 Cluj-Napoca, Romania; 4 Faculty of Biology and Geology, Babeş-Bolyai University, 1 Kogălniceanu, 400084 Cluj-Napoca, Romania

**Keywords:** Lacu Sărat Lake, balneo-climateric resort, salt lake pollution, heavy metals, human health risk, exposure, sediment contamination, risk assessment

## Abstract

Integrated study of both water and sediment in lakes provides important information regarding the human impact on the environment. The current work is focused on the correlation between age, source, composition, and degree of human intervention over the last 178 years and health impact of sediments from Lacu Sărat Lake (Romania), one of the most important balneo-climateric resorts in the country. The novelty relies on the fact that this is the first time the temporal patterns of metal contamination and the human health effects associated with the metal exposure from sediment core samples have been assessed. The sediment contamination status was determined by evaluating several indices, such as the enrichment factor, geo-accumulation index, metal pollution index, and potential ecological risk index, etc. Results showed a significant accumulation of Cd, Cr, As and Ni and a major contribution of Pb, Zn, Cd, Hg, Cr as well as Cu to the potential acute toxicity. The sediment quality guidelines emphasized a risk concerning the life and proper development of benthic organisms in Lacu Sărat Lake. Moreover, the incidental ingestion lifetime carcinogenic risk values for As and Cr suggest a potential risk of developing cancer. A strong human impact was observed especially between 1950 and 1990, which can be attributed to the rapid economic growth and intensive industrial development strategies pursued by the communist political regime in Romania.

## 1. Introduction

Aquatic ecosystems include heavy metals from both anthropogenic sources and natural rock weathering processes [1]. Urban, industrial and agricultural activities have significantly influenced the amount of heavy metals deposited in the environment and have disrupted their normal biogeochemical cycles [2,3]. In aquatic settings, sediments act as a sink for heavy metals [4]. Heavy metals are quickly absorbed by sediments despite their poor solubility in water. As a result, their transit through the aquatic systems is predominantly influenced by the texture and organic matter content of sediments. Physical, chemical, hydrological, and hydraulic variables are principally in charge of controlling the geographical distribution and accumulation of metals in sediments [5,6,7]. Riverine and lacustrine sediments are particularly vulnerable to pollution by heavy metals [8]. On the other hand, lake sediments represent archives of past and present changes in natural and anthropogenic contaminant input, providing significant information about human activities and their impact on the environment [9,10].

Heavy metals, especially As, Cd, Cr, Cu, Pb, Hg, Ni, and Zn, are listed as Priority Pollutants in the Clean Water Act program of The United States Environmental Protection Agency (USEPA) [11]. While some elements are critical micronutrients for life, others such as Cd, Cr, and Pb, are hazardous even in low quantities [12]. The persistent nature of these compounds is linked to their toxicological and ecotoxicological effects and their influence on human health [13]. According to nature, distribution and sources, each aquatic area has a distinct metal pollution pattern [1]. Estimating the level of contamination and its effects on human health is mandatory for locating and controlling the main sources of pollution. Numerous indicators, guidelines, and health risk assessment quotients can be used to gather important geochemical data for managing metals content in the environment [14]. Risk analyses are frequently used to evaluate the possible harm that metals pose to the ecosystems and human health, playing a significant role in regulatory decisions. A particular interest should be given to the pollution status of bathing locations (lakes, rivers, and coastal areas) since they account for more than 50% of international tourism [15,16]. The prevention and management of contamination in these types of locations must also take into account the ecological and health problems that they pose [17]. 

The environment is frequently exposed to metals through mixes. Considerable environmental damages were registered during the socialist era throughout the whole of Eastern Europe [18]. In Romania, only a few studies have been published in the field of paleolimnology [19], with most of the lake sediment records concentrating on the Carpathians area [20,21] or on the Danube Delta [22]. Salt lakes from the Romanian Plain have been studied regarding their origin, chemical composition, or environmental status [23,24]. However, there is still little knowledge available on the threat that combined metal pollution in Lacu Sărat Lake poses to the environment and human health.

Therefore, the present work is the first study investigating the composition, temporal distribution, potential sources, and ecotoxicological risk of trace metals in sediments from Lacu Sărat Lake, considered one of the most important balneo-climateric resorts in Romania. The aim of this study is to: (i) determine the sediment contamination status in the Lacu Sărat Lake area over the last 178 years; (ii) assess the contamination degree using pollution indices; (iii) assess the human health risk based on specific doses/quotients; and (iv) evaluate the relation between identified metals and possible sources using multivariate statistical analysis. The results obtained showed that the sediments in Lacu Sărat Lake were slightly polluted with heavy metals. 

## 2. Materials and Methods

### 2.1. Study Area

**Figure 1:**
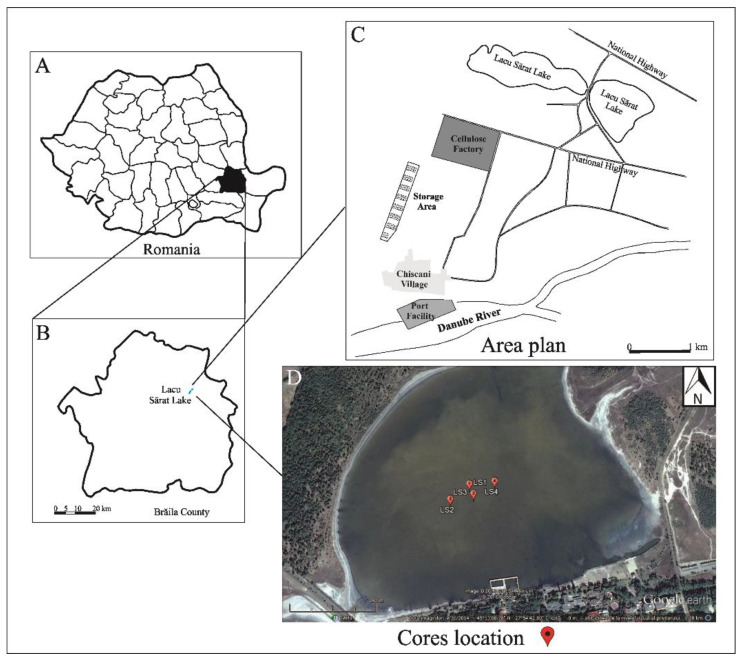
Map of the Lacu Sărat Area (Brăila County, Romania): (**A**) Brăila county location in Romania; (**B**) Lacu Sărat Lake location in Brăila county; (**C**) Simplified plan of Lacu Sărat Lake area; (**D**) Sediment cores location (Google Earth Map).

Lacu Sărat is located in the south-eastern part of Romania (45°13′11.67′′ N, 27°54′ 32.44′′ E), 6 km south-west of the town of Brăila, at 25 m above sea level, in the territory of Chiscani locality (Figure 1). The lake has 2 compartments (which communicate through a channel) and is known for its natural curative effects due to the salty water rich in sulphate, chlorine, sodium, magnesium, and calcium, the sapropelic mud and the stimulating steppe bio-climate [24]. It is a shallow lake with a maximum water depth of 2 m and has an area of 172 ha, in a region of Neogene–Quaternary age deposits composed of clays, gravels, and sands [25]. The hydrological basin consists of alluvial deposits (silty clays and sands) and loess and is covered by a 40 cm layer of sulphurous mud with 41% mineral content and 39% organic matter [26].

From a touristic point of view, the resort has been known since 1875 for the therapeutic qualities of its water and sapropelic mud, used for various affections, such as degenerative rheumatic diseases, gynecological, dermatological, and endocrine diseases, or for disorders of the peripheral nervous system. However, during the last few decades, the lake’s ecosystem has suffered from pollution due to urban, agricultural, and industrial development. The main industrial activities with a significant environmental impact in the studied area were those represented by Chiscani Cellulose and Paper Factory and Chemical Plant (starting from 1959) and by Chiscani Thermoelectric Power Plant (operating from 1973). In this regard, the construction of an industrial harbour began between 1957 and 1960 on the Danube River bank, in order to facilitate the transportation of raw materials supplied by Czechoslovakia and East Germany [27]. By the end of the 1950s, the water exchange between the two compartments of the lake was interrupted by the construction of a dike, the DN2b national road, and an industrial railroad which connected Lacu Sărat train station with the Chiscani industrial area [26]. Over time, all these anthropogenic activities affected the geochemical composition of the lake sediments and water.

### 2.2. Sampling and Analysis

The bathymetry of the lake has been investigated in the literature and the sampling location was chosen in order to avoid the areas disturbed by touristic activities or sapropelic mud exploitation. Thus, 4 sediment cores with different lengths (LS1—49 cm, LS2—40 cm, LS3—33 cm, and LS4—37 cm) were collected in November 2015 from the central part of Lacu Sărat Lake (water depth~55 cm) using a gravity corer (Figure 1). Samples were sliced at an interval of 1 cm and kept at −4 °C in self-sealed polyethylene bags in order to prevent contamination. In total, 159 sediment samples were further processed and investigated. Samples were analyzed for major and trace elements to assess the pollution status. 

^210^Pb is a natural radionuclide from the ^238^U decay chain, with a half-life of 22.30 years, and is widely used in dating different environments, such as ice, corals, or lake sediments [28,29,30]. The LS1 core was dated by the ^210^Pb radionuclide dating method and used to obtain the age-depth profile, while LS2 was used for geochemical analysis [31,32]. The ^210^Po sources were then measured using an ORTEC Soloist alpha-spectrometric system with PIPS detectors, having an active surface of 900 mm^2^ and a resolution better than 19 keV. The CRS model (Constant Rate of ^210^Pb Supply) developed by Appleby and Oldfield in 1978 was used to obtain the age-depth model for the sediment samples and calculate the sedimentation rates [33,34]. 

Heavy metal content from the LS2 core were analyzed at the Bureau Veritas Commodities Canada Ltd. (AcmeLab, Vancouver, BC, Canada). Concentrations were measured by inductively coupled plasma mass spectrometry (ICP-MS) for trace element determination and by inductively coupled plasma emission spectroscopy (ICP-OES) for major oxide analysis. Quality assurance/quality control procedures were used to ensure high quality data. Five types of control standards (STD GS311-1, STD GS910-4, STD DS10, STD OREAS45EA, and STD SO-19) were inserted to monitor analytical accuracy. Measurements were performed using LF200, AQ200, and TC000 methods. More details on accuracy and precision can be found at the following link: http//:www.acmelab.com (accessed on 5 April 2016). In total, 58 elements were analyzed for each sediment sample; however, in the current study only 12 chemical elements (Al, As, Cd, Co, Cr, Cu, Fe, Hg, Mn, Ni, Pb, and Zn) have been taken into account.

### 2.3. Pollution Indices

Generally, heavy metals are adsorbed on particles and are deposited at the bottom of sediments over time, with chronologically newer layers being deposited above the older ones [35]. A variety of evaluation indices such as contamination factor (C_f_), degree of contamination (C_d_), sediment enrichment factor (SEF), geo-accumulation Index (I_geo_), metal pollution index (MPI), pollution load index (PLI), and potential ecological risk index (PERI), were used to quantify the degree of metal contamination in sediments assessing the potential health risks. All the methodology regarding the determination of the above-mentioned indices can be found in the Supplementary Materials—Section SA. As regional geochemical background values were not available, we took the average shale values from [36] into consideration for the calculations.

### 2.4. Human Health Risk Assessment

In this study, the USEPA models were employed in order to estimate human exposure, potential human health risks, and carcinogenic and non-carcinogenic risks in relation to the metal contamination of sediments in Lacu Sărat area. The exposure was assessed using the average daily doses (ADDw), lifetime average daily doses (LADDw), hazard quotients (HQs), and hazard indices (HI). Exposure doses were calculated assuming a potential ingestion and a dermal absorption of the contaminated sediments (based on USEPA protocols) and were further compared with the USEPA reference doses (RfD) [37,38]. Furthermore, two scenarios were taken into consideration, namely the exposure of a child and of an adult. The equations used to estimate the exposure doses are presented in the Supplementary Materials—Section SA.

### 2.5. Statistical Analysis

Correlations among metal concentrations were tested through Pearson’s coefficient, setting the statistical significance at *p* < 0.05. Principal component analysis (PCA) and hierarchical agglomerative cluster analysis (HCA) were applied on multivariate data derived from the chemical investigation using IBM SPSS Statistics© Version 20.0 software (IBM SPSS Inc., Armonk, NY, USA). PCA represents a pattern recognition technique that enables a reduction of data and provides a description of a given multi-dimensional system by means of linear combinations called principal components (PCs) [39]. HCA reveals specific linkage between cases or variables, indicating similarities or dissimilarities regarding clusters [40].

## 3. Results and Discussions

### 3.1. ^226^Ra and ^210^Pb Concentrations

**Figure 2:**
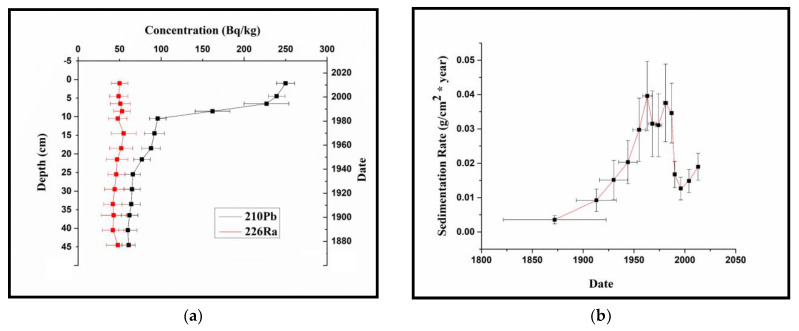
^210^Pb and ^226^Ra concentrations (**a**) and sedimentation rate (**b**) in Lacu Sărat Lake.

As can be seen in Figure 2, the ^210^Pb and ^226^Ra concentrations reach an equilibrium at the depth of 45 cm, which gives the lower limit of the dating interval. The activity concentration of ^210^Pb (supported) does not follow an exponential decrease, which clearly indicates a non-uniform sedimentation and the necessity for use of the CRS model for dating the sediment.

### 3.2. Age Depth Model of the Sediment Cores

**Figure 3:**
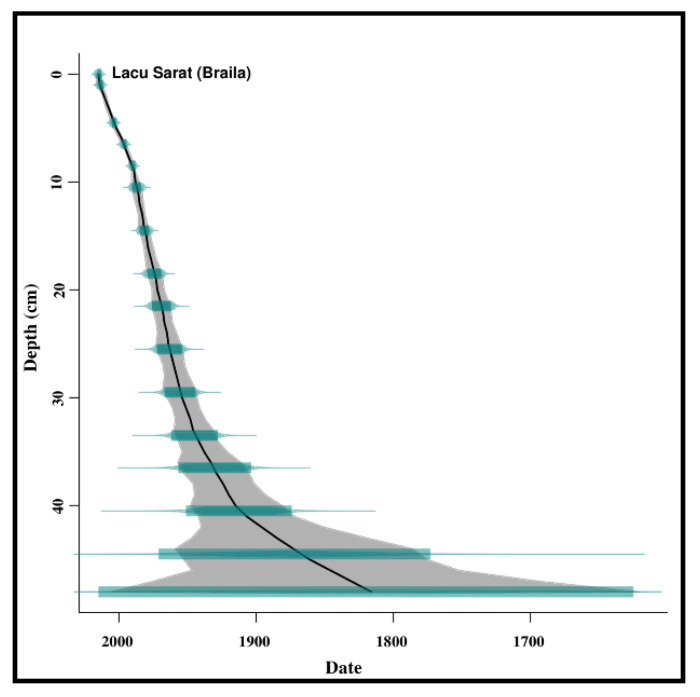
Age-depth model of the sediments from Lacu Sărat Lake. It can be noticed that the deepest part of the investigated sediment cores (48 cm) corresponds to the year 1836.

A direct relation between sediment ages and depth can be seen in Figure 3. The deepest part of the studied sediment cores (48 cm) corresponds to the oldest age (1836 AD). The first 54 years (from 2014 to 1960) can be correlated with the upper 30 cm of the sediment cores, while the other 124 years (from 1960 to 1836) correspond to the following 18 cm. 

### 3.3. Sedimentation Rates

Lacu Sărat Lake exhibits a growing sedimentation rate for 127 years, from 0.004 ± 0.001 g/cm^2^y in 1836 to 0.038 ± 0.010 g/cm^2^y in 1963, followed by a decrease to 0.031 ± 0.009 g/cm^2^y in 1974 (Figure 2b). The increased input of sediments between 1950 and 1990 is both a result of the administrative measures adopted by the communist political regime (which accelerated deforestation and industrialization in the Lacu Sărat Lake area), and a consequence of significant hydrologic events followed by soil erosion. The highest sedimentation rate was recorded between 1956 and 1981 (0.038 ± 0.011 g/cm^2^y), followed by a second peak between 1982 and 1986 (0.035 ± 0.009 g/cm^2^y). Between 1987 and 1996, the sedimentation rate decreased from 0.035 ± 0.009 g/cm^2^y to 0.013 ± 0.003 g/cm^2^y, followed by a rapid increase from 0.013 ± 0.003 g/cm^2^y in 1997 to 0.019 ± 0.004 g/cm^2^y in 2014. 

### 3.4. Temporal Distribution (Historical Changes) of Heavy Metals

**Figure 4:**
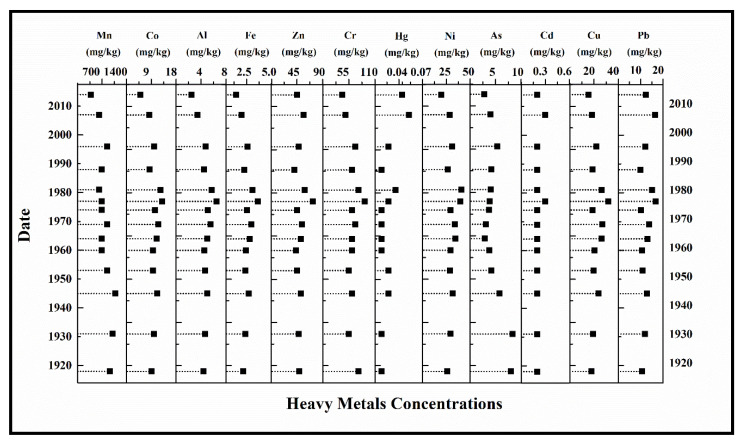
Temporal variation of heavy metals in sediment samples from Lacu Sărat Lake. Similar variation patterns can be observed for the following groups of elements: Cu-Pb, Zn-Cr-Ni-Hg, and Co-Al-Fe.

The heavy metal concentrations measured in the LS2 sediment core exhibited significant variation during the last century (Figure 4). Similar variation patterns have been observed for the following groups of elements: Cu-Pb, Zn-Cr-Ni-Hg, and Co-Al-Fe. Important heavy metals uptakes can be distinguished in the periods of 1943–1948, 1973–1983, and 2003–2006. Both the intensification of human activities and high amounts of precipitations influenced the fluctuations of elements in lacustrine sediments. The results for the heavy metals analysis in the sediment samples from Lacu Sărat Lake were compared with the maximum permissible limits provided by the sediment quality standards established by environmental regulatory authorities in Romania, Canada, and the United States of America (Table S1). 

### 3.5. Geo-Accumulation Index

The sources of pollution were determined through the normalization of geo-accumulation indices to the reference elements. The results for the geo-accumulation index (I_geo_) are shown in Table 1. The negative values indicate the fact that the lake sediments are not polluted with Zn, Al, Cu, Pb, Cd, Co, and Fe. The values of I_geo_ showed low to moderate pollution for Mn (between 1918 and 1945), Cr (between 1918 and 1996), As (in the periods of 1953–1988 and 2007–2014), and Ni (in 1945, between 1964 and 1969 and between 1977 and 1981). Moreover, in case of As, I_geo_ revealed a moderate pollution level between 1918 and 1945 and also in 1996.

### 3.6. Contamination Factor

**Figure 5:**
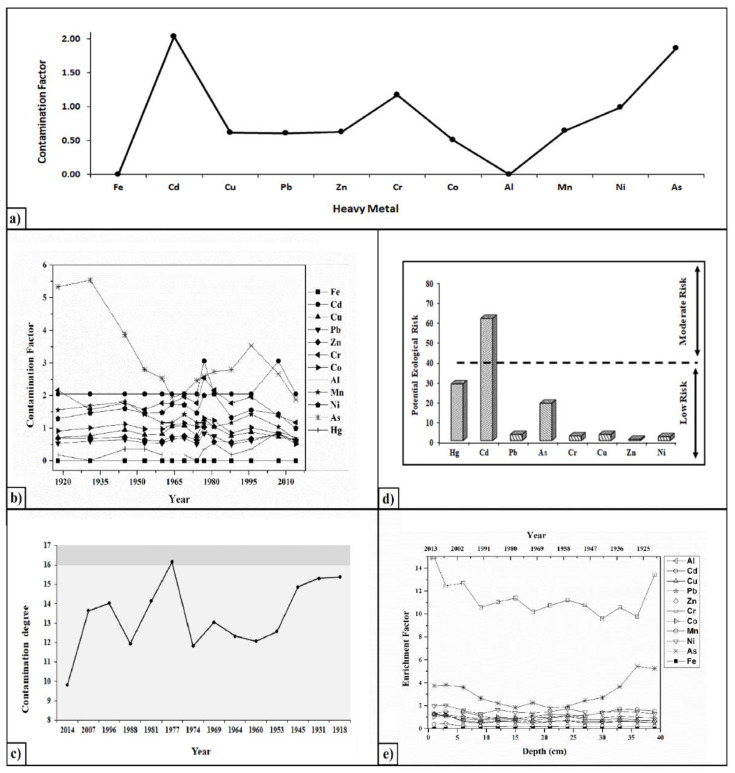
Contamination factor in surface sediment samples (**a**), temporal fluctuation of the contamination factor (**b**) and temporal variation of contamination degree between 1918–2014 (bright grey—moderate degree of contamination; dark grey—considerable degree of contamination) (**c**), potential ecological risk of investigated elements (**d**), enrichment factor of investigated heavy metals (**e**).

The variation of the contamination factor was calculated for the superficial sediments based on the concentrations of Fe, Cd, Cu, Pb, Zn, Cr, Co, Al, Mn, Ni, and As (Figure 5a). Moreover, the temporal fluctuations of the heavy metals show low contamination values for Fe, Pb, Al, and Hg (less than one) and moderate for Cr (between one and three) (Figure 5b). Mn and Ni present similar trends regarding the contamination factors, exhibiting moderate values between 1918 and 2007 and lower ones between 2008 and 2014. In general, Zn displays low levels of contamination, except for the period between 1975 and 1980, when moderate contamination occurred. Sediment samples are also characterized by a moderate contamination with Co (in the periods of 1931–1945, 1964–1981, and 1989–2000) and Cu (in the periods of 1964–1969 and 1977–1981). Cd shows moderate contamination, except between 1975 and 1977 and between 1997 and 2007, when the contamination factor exhibited considerable values. Likewise, the contamination factor for As also presented moderate values, except in the periods of 1918–1945 and 1989–1996, when its degree was considerable.

### 3.7. Degree of Contamination, Modified Degree of Contamination, Metal Pollution Index and Pollution Load Index

Values of both modified degree of contamination (mCd) and pollution load index (PLI) indicate no significant pollution in the superficial sediments of Lacu Sărat Lake (Table S2). On the other hand, the results for the overall degree of contamination (Cd) show a moderate level of pollution for the superficial sediments. The temporal variation of the degree of contamination from Lacu Sărat Lake sediments shows moderate values, except for the period between 1975 and 1977, when considerable contamination occurred (Figure 5c).

### 3.8. Potential Ecological Risk

An ecological risk assessment was conducted in order to identify the potential ecological risks associated with toxic metals contents in sediments from Lacu Sărat Lake. The calculated potential ecological risk index for each element is presented in Figure 5d. As it can be seen, the potential ecological risk index decreased in the following order: Cd (61.20) > Hg (28.400) > As (18.600) > Cu (3.100) > Pb (3.000) > Cr (2.300) > Ni (1.900) > Zn (0.600). Among them, Cd is at a moderate risk, while the other metals present a low degree of potential ecological risk. The average PERI indicate that sediments from Lacu Sărat Lake present a low ecological risk. 

### 3.9. Enrichment Factor

The enrichment factors for heavy metals in sediments from Lacu Sărat Lake are presented in Figure 5e. As it can be seen, the values of the enrichment factor for most of the studied heavy metals are lower than two, indicating a natural enrichment (depletion to minimal enrichment). Furthermore, most of the amounts of Cr and As come from the surrounding anthropogenic activities. These results suggest a significant accumulation of Cr and As in lake sediments that may need more attention to be paid for the risk assessment. The sequence of Ef for heavy metals in the sediments is in the following order: Cr > As > Ni > Co > Cu > Pb > Zn > Cd > Fe > Al. This indicates that Cr was more abundant when compared with the other metals, whereas Al and Fe had the lowest appearance. 

### 3.10. Comparison between SQGs, TEL, TEC, ERL, PEL, PEC, ERM

Excessive accumulation of heavy metals in sediments may induce ecotoxicological effects for both freshwater and aquatic ecosystems. SQGs provide information for evaluating the risks posed to sediment-dwelling organisms by sediment-associated contaminants. Cu, Pb, Cd, Zn, As, and Hg values are lower than TEL, TEC, ERL, PEL, PEC, and ERM in all samples, showing that the sediments can rarely pose a risk for aquatic biota regarding these types of heavy metals pollution (Table 2). The mean PEL and ERM quotients have been applied for the measured heavy metals in order to determine the possible biological effects of combined toxicant groups. Based on the m-PEL-Q the Lacu Sărat Lake area can be classified as having low-medium priority, while m-ERM-Q showed that this mixture of contaminants has a 49% probability of toxicity for the aquatic biota. The SQGQ results emphasize that the environment in the Lacu Sărat Lake area presents a risk concerning the life and proper development of benthic organisms.

### 3.11. Toxic Units (TUs) and Toxic Risk Index (TRI)

**Figure 6:**
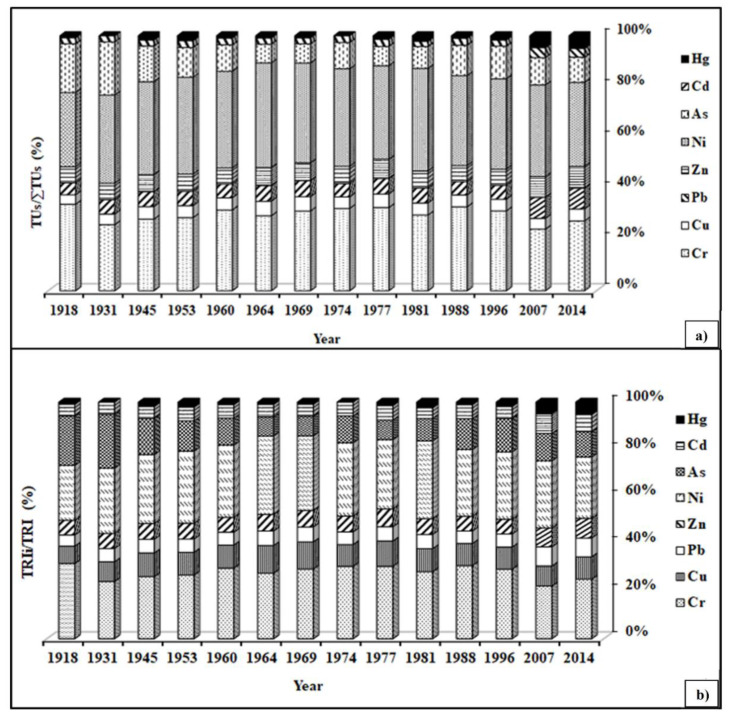
Temporal distribution of TUs/∑TUs ratio of investigated heavy metals (**a**) and of toxic risk index of investigated heavy metals (**b**).

The mean levels of toxic units (TUs) for all the heavy metals investigated in the sediment samples from Lacu Sărat Lake varied from 0.020 to 1.130, following this sequence: Ni > Cr > As > Zn > Pb > Cu > Cd > Hg. The Tus/∑Tus ratio revealed the individual contribution of each of the eight heavy metals to their total potential acute toxicity in the sediment samples (Figure 6a), indicating that in general, Ni, Cr, and As had a higher potential toxicity for the ecosystems in the Lacu Sărat Lake area. Thus, proactive actions are essential in order to monitor and reduce the metal contamination induced by anthropogenic activities in the nearby area. The input of Hg in the potential acute toxicity was the lowest (1–2% on average) compared with the other heavy metals investigated. The temporal distribution of the toxic units indicates a significant increase in the contribution of Pb, Zn, Cd, and Hg to the potential acute toxicity between 2007 and 2014, most probably due to the intensification of urban traffic, whereas for Cr, Cu and Ni the highest values were observed in the period of industrialization (between 1964 and 1981). As contribution was higher mainly between 1918 and 1931. However, attention should also be paid to Cd, Hg, Pb, and Zn because of their high contributions to the total toxic risk index in the last decade (Figure 6b). A strong correlation (R^2^ = 0.996) can be noticed between TRI and ∑TUs, suggesting that the information provided by these two indices shows actual relevance for the estimation of the general ecotoxicology (Figure S1). 

### 3.12. Human Health Risk Assessment

The results obtained regarding the human health risk assessment estimated for the dermal contact and the incidental ingestion of sediments from the Lacu Sărat Lake area are presented in Table 3 and Table S3. The ADDs and LADDs calculated for dermal contact of adults with the lake sediments do not exceed RfDs, except for As and Cr. The ADDs of Cd, As, and Cr calculated for the scenario regarding the dermal contact of children with the lake sediments are higher than the reference values established by USEPA. The scenarios of incidental sediment ingestion for both children’s and adults’ exposure indicate no exceeding of RfDs. In general, the hazard quotients estimated for both dermal and incidental ingestion scenarios are lower than the values of RfDs, except for the case of As and Cr dermal contact exposure of adults. Furthermore, the non-carcinogenic risks for both dermal and ingestion scenarios were lower than one, showing that it is not likely that the measured contaminant levels in the sediment samples will pose a risk to the potentially exposed population groups. However, the hazard indices were higher in the case of children as compared to adults, indicating that the health risks related to heavy metal exposure in sediments are higher for children. Nevertheless, the dermal lifetime carcinogenic risk of As is lower than 10^−6^, indicating that the dermal absorption might not be a matter of concern for the exposed population [47]. On the other hand, the incidental ingestion lifetime carcinogenic risks are lower than 10^−6^, except for As in the scenario of adults’ exposure and for Cr in both adults’ and children’s exposure scenarios, where the LCR values are between 10^−6^ and 10^−4^, suggesting potential cancer risks [48]. The cumulative non-carcinogenic risks are lower than one, enhancing the fact that the heavy metals exposure is lower than the reference values and that it is unlikely to result in adverse effects for both children and adults. In general, the cumulative carcinogenic risks are lower than 10^−6^, showing that there are no significant risks for both adults and children of developing cancer during their lifetime due to the heavy metal exposure from dermal contact or ingestion of sediments from the Lacu Sărat Lake area. An exception in this regard is for As in the scenario of adults’ exposure and for Cr in both adults’ and children’s exposure scenarios, where the cumulative carcinogenic risks are between 10^−6^ and 10^−4^, indicating potential risks.

### 3.13. Statistical Analysis of the Heavy Metals Content in Sediments

**Figure 7:**
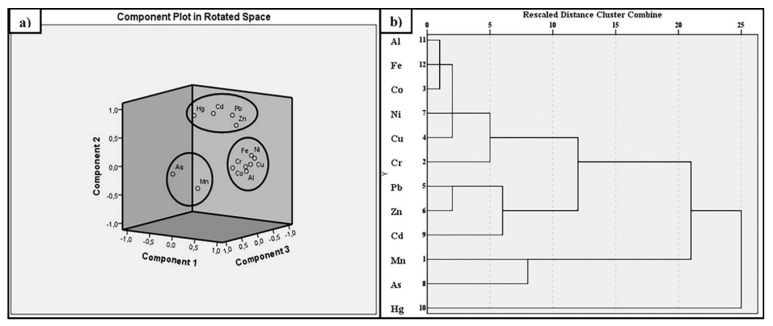
Principal component analysis (**a**) and multivariate hierarchical clustering (**b**) of the investigated heavy metals from Lacu Sărat Lake sediments.

The descriptive statistics (Table S4) and Pearson’s correlation coefficient (Table S5) were used to identify the basic components and relations between the heavy metals from Lacu Sărat Lake sediments. PCA was used to diminish the number of variables to only three principal components, which contribute 91.70% of the data variance (PC1—52.380%, PC2—23.750%, and PC3—15.570%). PC1 included Fe, Al, Co, Cu, Ni, and Cr; PC2 was dominated by Cd, Pb, Hg, and Zn; and PC3 consisted of Mn and As (Figure 7a). Al and Fe are usually associated with natural sources, namely rock-forming minerals, while Cd, Pb, Hg, and Zn indicate an uptake due to anthropogenic perturbation such as deposition from agricultural activities or traffic emissions (diesel, oil combustion, tire, and brake abrasion) [40,49]. The high influx of Zn in PC1 and PC2 highlights almost equal contributions from natural and anthropogenic sources (Table S6). It can be noticed that As had a slightly unique anthropogenic origin. HCA differentiated the samples into three clusters based on the investigated metals, which were also correlated with the three principal components from the PCA (Figure 7b). The first cluster had two sub-groups: Al-Fe-Co-Ni-Cu-Cr formed one sub-group, while the second sub-group consisted of Pb-Zn-Cd. The second cluster consisted of Mn and As, both linked to anthropogenic activities’ input [50], whereas the last cluster was represented by Hg. Al, Fe, and Co are commonly associated with the lithologic intrusions, weathering and mineralogical structure dominated by clays and silt [51]. In general, Pb, Zn, and Cd represent markers for diesel or oil combustion (alkyl-lead additives in gasoline and use of leaded gasoline before prohibition), tire and brake abrasion and can be correlated with traffic emissions [52]. As, Cd, Cu, Zn, Pb, and Ni can also originate from manure or agricultural activities, such as the use of phosphate fertilizers and pesticides [40]. The limited water exchange in the lake due to the construction of the dike could have accelerated the metal pollution. The input of Cu can be associated with the antifouling paints used in the industrial harbour or with the boats abandoned on the lake [53]. Hg, Cd, Zn, Pb, and Cu can also be generated through industrial discharges and unfiltered emissions from the Chiscani paper making industry and Galați iron and steel industry [54].

### 3.14. Sources of Metal Contamination in Lacu Sărat Area

Activities related to agriculture, tourism, chemical and electric power industries are mainly responsible for the environmental impact on the Lacu Sărat area. The anthropogenic footprint associated with the content of inorganic and organic matter in the lake sediments describes the historical changes that occurred in this area and also exhibits a scenario regarding the evolution of environmental quality during the last century. Heavy metal concentrations measured in the sediment samples increased sharply mainly between 1950 and 1990, and this can be attributed to the rapid economic growth and intensive industrial development plan implemented using the strategies formulated by the communist political regime in Romania. The input of Cu, Pb, and Zn can be associated with the heavy traffic induced by the construction of the national road near the lake. Brake pad wear debris and tire wear material are enriched in Cu and Zn, together with a lower amount of Pb. Thus, traffic contributes significantly to the metal pollution of roadway runoff waters through engine oil spills, exhaust emissions, and rain drainage [55]. Ni, Cu, and Cr are usually correlated with oil spills [56], which, in this case, could have been caused either by the nearby traffic or by point sources from the Chiscani industrial area. Urban storm water runoff is also rich in heavy metals [40]. The large steelworks in Galaţi could have also contributed to the general heavy metal loading between 1960 and 1990, through long-distance dispersion and emissions of high concentrations of Al, As, Co, Cr, Fe, Mn, Zn, and Ni. As and Hg are atmophile elements; thus, dry and wet atmospheric deposition either from Galaţi or Chiscani industrial areas could have also increased their concentrations in the environment. Meena et al. [57] showed that the inadequate management of batteries and ligands can generate Pb and Cr contamination. Therefore, the storage platform for large batteries/ligands from the Chiscani industrial complex could have also contributed to the total input of Pb and Cr. Moreover, surface runoff from agricultural activities on the terrains located near the lake could have contributed to the enrichment in heavy metals originating from pesticides or fertilizers. For example, Nicholson et al. [58] reported that herbicides could release high quantities of As into the environment, while phosphate, nitrogen, and lime fertilizers can generate important amounts of Cd, Cr, Cu, Ni, Pb, and Zn. Furthermore, livestock manure from pig farms and poultry units in the nearby area could have had a significant input in the content of Zn, Cu, Ni, Pb, and Cr. Domestic sewage discharges from touristic activities can also produce metal contamination [40]. The development of balneary tourism intensified in the area during the last two centuries, since Lacu Sărat Lake holds one of the most valuable natural therapeutic resources in Romania, namely the unique curative mud. Thus, the tourism exploitation may have also released certain amounts of Zn, Cu, Ni, Cr, and Pb into the environment, which were later deposited in the bottom sediments of the lake. 

## 4. Conclusions

The present study represents the first comprehensive eco-toxicological risk assessment in Lacu Sărat Lake, Romania during the last century. Our results reveal the pollution status and the potential risks induced by the metals input in the lake sediments. The anthropogenic footprint associated with the heavy metal content in the lake sediments exhibits a scenario regarding the temporal evolution of environmental quality and the environmental changes that have occurred in this area due to human impact during the last 178 years:

- The results highlight a higher input of metals between 1950 and 1990, attributed to the rapid economic growth and intensive industrial development from the communist regime.
- The variation patterns identified through PCA and HCA analysis show clear correlations among the investigated metals, highlighting a strong dependency with their sources (lithogenic and anthropogenic).
- Based on the obtained results the superficial sediments from Lacu Sărat Lake are slightly polluted with heavy metals. Among them, Cd, Cr, As, and Ni pose a moderate risk despite considerable contamination occurring between 1970 and 1985. The results also suggest a significant accumulation of Cr and As that may need more attention to be paid for the risk assessment.
- The temporal distribution of the toxic units indicates a significant contribution of Pb, Zn, Cd, and Hg to the potential acute toxicity between 2007 and 2014, whereas for Cr, Cu, and Ni the highest values were observed in the period of industrialization. The values of the toxic risk index show that Ni, Cr, and As had the higher potential toxicity for the ecosystems in the Lacu Sărat Lake area.
- Sediment quality guidelines were also applied, and the results emphasize that the environment in Lacu Sărat Lake presents a risk concerning the life and proper development of benthic organisms.
- Lacu Sărat Lake has a balneo-climateric function and represents one of the most valuable touristic attractions in Romania. Therefore, multiple scenarios were used in order to assess the health risk for both adults and children considering the dermal contact and the incidental ingestion of sediments from Lacu Sărat Lake. These scenarios indicate that it is unlikely that the measured contaminants levels in the sediment samples will pose acute adverse effects on the potentially exposed population groups. On the other hand, the incidental ingestion lifetime carcinogenic risks for As in the scenario of adults’ exposure and for Cr in both adults’ and children’s exposure scenarios suggest a potential risk of developing cancer during their lifetime.

The present study offers the possibility to compare the quality status and the risks imparted by metals content in various lake sediments on regional and global scales. However, continuous efforts are still needed to conduct a systematic investigation and more similar surveys for a better understanding of the spatial distribution of metals. Furthermore, proactive actions are required to monitor and control the heavy metals input caused by nearby anthropogenic sources in order to prevent further quality degradation of the lake. Considering the pollution status and the function of the lake, it is mandatory to recommend the implementation of specific policies, regulations, and standards for balneo-climateric resorts. The results of the current study represent a baseline for future research on anthropogenic impacts in this region and the methods applied can also be used for pollution assessment in other areas. 

## Figures and Tables

**Table 1:** Geo-accumulation index (I_geo_) of the investigated metals for the 1918–2014 period.

Element		I_geo_ Mn	I_geo_ Zn	I_geo_ Al	I_geo_ Cu	I_geo_ Pb	I_geo_ Cr	I_geo_ As	I_geo_ Cd	I_geo_ Ni	I_geo_ Co	I_geo_ Fe
	Year											
**2014**		−1.220	−1.240	−15.550	−1.270	−1.290	−0.350	0.310	−2.900	−0.600	−1.560	−15.130
**2007**		−0.540	−0.900	−15.100	−1.030	−0.860	−0.130	0.830	−2.320	−0.070	−0.850	−14.690
**1996**		−0.080	−1.150	−14.630	−0.780	−1.300	0.380	1.240	−2.900	0.050	−0.570	−14.300
**1988**		−0.370	−1.380	−14.720	−0.980	−1.580	0.230	0.900	−2.900	−0.180	−0.820	−14.480
**1981**		−0.540	−0.850	−14.360	−0.520	−0.990	0.520	0.870	−2.900	0.440	−0.290	−14.040
**1977**		−0.370	−0.540	−14.180	−0.240	−0.840	0.760	0.790	−2.320	0.410	−0.210	−13.830
**1974**		−0.370	−1.240	−14.530	−0.990	−1.560	0.230	0.720	−2.900	−0.040	−0.540	−14.320
**1969**		−0.080	−0.980	−14.420	−0.480	−1.110	0.380	0.460	−2.900	0.190	−0.380	−14.110
**1964**		−0.370	−1.030	−14.570	−0.510	−1.210	0.230	0.370	−2.900	0.200	−0.450	−14.190
**1960**		−0.370	−1.310	−14.710	−0.880	−1.500	0.230	0.760	−2.900	−0.020	−0.630	−14.410
**1953**		−0.080	−1.240	−14.660	−0.930	−1.460	0.060	0.900	−2.900	−0.050	−0.630	−14.410
**1945**		0.270	−1.030	−14.550	−0.670	−1.220	0.230	1.370	−2.900	0.090	−0.420	−14.230
**1931**		0.160	−1.150	−14.670	−0.970	−1.320	0.060	1.880	−2.900	−0.040	−0.580	−14.430
**1918**		0.050	−1.120	−14.730	−1.070	−1.500	0.520	1.830	−2.900	−0.220	−0.720	−14.550

Light grey color shows low pollution level and dark grey indicates moderate pollution.

**Table 2:** Heavy metals content and values of SQGs, TEL, TEC, ERL, PEL, PEC and ERM.

Element		Mn (mg/kg)	Cr (mg/kg)	Co (mg/kg)	Cu (mg/kg)	Pb (mg/kg)	Zn (mg/kg)	Ni (mg/kg)	As (mg/kg)	Cd (mg/kg)	Hg (mg/kg)	Al (mg/kg)	Fe (mg/kg)
	Depth												
**1 cm**		387.3	41.060	5.100	15.500	12.300	45.000	19.800	2.800	0.200	0.040	2.510	1.460
**3 cm**		619.7	47.910	8.300	18.300	16.500	57.00	28.500	4.000	0.300	0.050	3.430	1.990
**6 cm**		852.1	68.44	10.100	21.900	12.200	48.000	31.000	5.300	0.200	0.020	4.750	2.600
**9 cm**		697.1	61.60	8.500	19.000	10.000	41.000	26.400	4.200	0.200	0.010	4.470	2.290
**12 cm**		619.7	75.29	12.300	26.200	15.100	59.00	40.800	4.100	0.200	0.030	5.730	3.120
**15 cm**		697.1	88.98	13.000	31.700	16.700	73.00	39.900	3.900	0.300	0.020	6.500	3.610
**18 cm**		697.1	61.60	10.300	18.900	10.200	45.000	29.200	3.700	0.200	LDL	5.090	2.560
**21 cm**		852.1	68.44	11.500	26.900	13.900	54.00	34.200	3.100	0.200	0.010	5.490	2.970
**24 cm**		697.1	61.60	11.000	26.300	13.000	52.00	34.400	2.900	0.200	0.010	4.970	2.820
**27 cm**		852.1	61.60	9.700	20.400	10.600	43.000	29.500	3.800	0.200	0.010	4.510	2.420
**30 cm**		852.1	54.75	9.700	19.700	10.900	45.000	29.000	4.200	0.200	0.020	4.660	2.410
**33 cm**		1084.4	61.60	11.200	23.600	12.900	52.00	31.900	5.800	0.200	0.020	5.010	2.730
**36 cm**		1006.9	54.75	10.000	19.100	12.000	48.000	29.200	8.300	0.200	LDL	4.620	2.390
**39 cm**		929.5	75.29	9.100	17.800	10.600	49.000	25.800	8.000	0.200	0.010	4.430	2.200
**TEL ^a^**		**-**	**37.300**	**-**	**35.700**	**35**	**123**	**18**	**5.900**	**0.590**	**0.170**	**-**	**-**
**PEL ^b^**		**-**	**90**	**-**	**197**	**91.30**	**315**	**36**	**17**	**3.530**	**0.480**	**-**	**-**
**m-PEL-Q ^c^**		**-**	**0.700**	**-**	**0.110**	**0.140**	**0.160**	**0.850**	**0.270**	**0.060**	**0.040**	**-**	**-**
**ERL ^d^**		**-**	**80**	**-**	**70**	**35**	**120**	**30**	**33**	**5**	**0.150**	**-**	**-**
**ERM ^e^**		**-**	**145.0**	**-**	**390**	**110**	**270**	**50**	**85**	**9**	**1.300**	**-**	**-**
**m-ERM-Q ^f^**		**-**	**7.010**	**-**	**0.060**	**0.110**	**0.190**	**0.610**	**0.050**	**0.020**	**0.020**	**-**	**-**
**TEC ^g^**		**-**	**43.400**	**-**	**31.600**	**35.800**	**121**	**22.700**	**9.790**	**0.990**	**0.180**	**-**	**-**
**PEC ^h^**		**-**	**111**	**-**	**149**	**128**	**459**	**48.600**	**33.000**	**4.980**	**1.060**	**-**	**-**
**SQGQs ^i^**		**-**	**0.630**	**-**	**0.550**	**0.150**	**0.340**	**0.880**	**0.160**	**0.270**	**0.070**	**-**	**-**

^a^ TEL = threshold effect level below which adverse effects are expected to occur only rarely [41]. ^b^ PEL = probable effect level above which adverse effects are expected to occur frequently [41]. ^c^ m-PEL-Q = mean PEL quotient which accounts for the additive toxic effects of a mixture of chemicals [42]. ^d^ ERL = the effect range low below which adverse effects would be rarely observed [43]. ^e^ ERM = the effect range median above which adverse effects would frequently occur [43]. ^f^ m-ERM-Q = mean ERM quotient which highlights the probability of toxicity for biota [44]. ^g^ TEC = threshold effect concentration below which adverse effects are not expected to occur [45]. ^h^ PEC = probable effect concentration above which adverse effects are expected to occur more often [45]. ^i^ SQGQs = sediment quality guideline quotients [46].

**Table 3:** Human non-carcinogenic and carcinogenic risk of heavy metals from sediments considering accidental ingestion or dermal contact.

Risks Dermal Exposure	Receptor	Heavy Metal											
		Pb	Cd	As	Cu	Zn	Cr	Al	Ni	Mn	Hg	Fe	Co
**Dermal Hazard Quotient** **(HQ_dermal_)**	**Adult**	1.503 × 10^−2^	9.320 × 10^−1^	1.410	1.700 × 10^−3^	6.340 × 10^−4^	4.250	1.110 × 10^−5^	1.130 × 10^−1^	3.350 × 10^−1^	9.320 × 10^−4^	9.200 × 10^−6^	8.330 × 10^−2^
	**Children**	3.150 × 10^−2^	1.920 × 10^−3^	8.690 × 10^−2^	3.500 × 10^−3^	1.300 × 10^−3^	1.140 × 10^−1^	2.280 × 10^−5^	9.260 × 10^−3^	2.760 × 10^−2^	1.920 × 10^−3^	1.890 × 10^−5^	1.710 × 10^−1^
**Ingestion Hazard Quotient** **(HQ_ingestion_)**	**Adult**	1.960 × 10^−3^	1.190 × 10^−4^	5.410 × 10^−3^	2.180 × 10^−4^	8.120 × 10^−5^	7.080 × 10^−3^	1.420 × 10^−6^	5.770 × 10^−4^	1.720 × 10^−3^	1.190 × 10^−4^	1.180 × 10^−6^	1.070 × 10^−2^
	**Children**	6.760 × 10^−3^	4.110 × 10^−4^	1.860 × 10^−2^	7.510 × 10^−4^	2.790 × 10^−4^	2.440 × 10^−2^	4.880 × 10^−6^	1.980 × 10^−3^	5.910 × 10^−3^	4.110 × 10^−4^	4.060 × 10^−6^	3.670 × 10^−2^
**Non-carcinogenic Dermal Risk (HI_dermal_)**	**Adult**	1.050 × 10^−1^											
	**Children**	4.490 × 10^−1^											
**Non-carcinogenic Ingestion Risk** **(HI_ingestion_)**	**Adult**	2.800 × 10^−2^											
	**Children**	9.620 × 10^−2^											
**Lifetime—Dermal Carcinogenic Risk**	**Adult**	-	-	2.440 × 10^−7^	-	-	-	-	-	-	-	-	-
	**Children**	-	-	1.170 × 10^−7^	-	-	-	-	-	-	-	-	-
**Lifetime—Ingestion Carcinogenic Risk**	**Adult**	2.500 × 10^−8^	3.220 × 10^−7^	1.040 × 10^−6^	-	-	4.550 × 10^−6^	-	-	-	-	-	-
	**Children**	2.010 × 10^−8^	2.590 × 10^−7^	8.380 × 10^−7^	-	-	3.660 × 10^−6^	-	-	-	-	-	-
**Cumulative** **Non-Carcinogenic Risk**	**Adult**	1.330 × 10^−1^											
	**Children**	5.450 × 10^−1^											
**Cumulative** **Carcinogenic Risk**	**Adult**	2.500 × 10^−8^	3.220 × 10^−7^	1.290 × 10^−6^	-	-	4.550 × 10^−6^	-	-	-	-	-	-
	**Children**	2.010 × 10^−8^	2.590 × 10^−7^	9.560 × 10^−7^	-	-	3.660 × 10^−6^	-	-	-	-	-	-

Light grey color indicates limit exceeding.

## Data Availability

All data generated or analyzed in this study can be found in the article and in the electronic Supplementary Materials.

## Supplementary Materials

The following supporting information can be downloaded at: https://www.mdpi.com/article/10.3390/ijerph20021342/s1, Section S.A.: Methods for The Determination of Pollution Indices and Exposure Doses; Section S.B.: Results of The Metal Pollution Indices in Lacu Sărat Sediments; Section S.C.: Results of Statistical Analysis Applied on The Heavy Metals Content in Lacu Sărat Sediments; Table S1: Maximum permissible limits of heavy metals according to various legislations; Table S2: Values of Cd, mCd, MPI, and PLI for investigated heavy metals; Figure S1: Correlation between TRI and ∑Tus; Table S3: Exposure doses to heavy metals from sediments considering the accidental ingestion or dermal contact scenarios; Table S4: Descriptive statistics of investigated heavy metals; Table S5: Pearson correlation matrix of the investigated metals from Lacu Sărat Lake sediments; Table S6: Principal component analysis for selected heavy metals in lake sediments from Lacu Sărat Lake.
