# Supplementary material for: Combining Contamination Indices and Multivariate Statistical Analysis for Metal Pollution Evaluation during the Last Century in Lacustrine Sediments of Lacu Sărat Lake, Romania
Source: Int J Environ Res Public Health. 2023 Jan 11;20(2):1342. doi: 10.3390/ijerph20021342 (PMC9858634; doi:10.3390/ijerph20021342)
Supplement: Supplementary file 1 [file ijerph-20-01342-s001.zip › ijerph-2134769-supplementary.pdf]

International Journal of
Environmental Research and Public Health

MDPI

Supplementary Materials

Combining contamination indices and multivariate statistical analysis for metal pollution evaluation during the last century in lacustrine sediments of Lacu Sărat Lake, Romania

Iolanda-Veronica Ganea [1,2], Ramona Bălc [1,3,*], Robert-Csaba Begy [1,3], Ioan Tanțău [4] and Delia Maria Gligor [1]

S.A. Methods for The Determination of Pollution Indices and Exposure Doses

The contamination factor (C_f) is often used for pollution monitoring and assessment over a specific period for different substances [1]. The individual contamination factors are determined based on the following formula (1):

$$C_f = \frac{C_0^i}{C_n^i} \quad (1)$$

where: C_0^i is the concentration of metal in sediments and C_n^i is the background value for a specific metal. C_f is usually defined according to four classes as follows: $C_f < 1$ (low contamination factor), $1 \le C_f < 3$ (moderate contamination factor), $3 \le C_f < 6$ (considerable contamination factor), $C_f \ge 6$ (very high contamination factor) [2].

The overall degree of contamination (Cd) is calculated with the next Equation(2) [3]:

$$C_d = \sum_{i=1}^{9} C_f^i \quad (2)$$

where: C_f^i is the contamination factor obtained for each substance. Håkanson [4] classified the degree of contamination according to four categories: Cd < 8 (low degree of contamination), 8 ≤ Cd < 16 (moderate degree of contamination), 16 ≤ Cd < 32 (considerable degree of contamination) and Cd ≥ 32 (very high degree of contamination – serious anthropogenic pollution).

The Sediment Enrichment Factor (SEF) is an effective instrument for evaluating the magnitude of contaminants in the environment. Iron and aluminum are usually reference elements to distinguish natural from anthropogenic components [5]. SEF is expressed by the next Equation (3):

$$SEF = \frac{C_0/C_{ref}\ (\text{sample})}{B_n/B_{ref}\ (\text{background})} \quad (3)$$

where: C_0 is the concentration of the examined element in the sediment sample, C_{ref} is the concentration of the reference element in the sediment sample, B_n is the concentration of the examined element in the background, and B_{ref} is the concentration of the reference element in the background. Five contamination categories are recognized based on SEF values: SEF < 2 (deficiency to minimal enrichment), 2 ≤ SEF < 5 (moderate enrichment), 5 ≤ SEF < 20 (significant enrichment), 20 ≤ SEF < 40 (very high enrichment), SEF ≥ 40 (extremely high enrichment) [6].

The Geo-accumulation Index (I_{geo}) was employed to separate the anthropogenic influences on the sediment samples from the natural influences and was calculated for different metals according to Müller [7] as follows (4):

$$I_{geo} = \log_2 \frac{C_0}{k \times B_n} \quad (4)$$

where: C_0 represents the metal concentrations in the sediment samples, B_n is the background content, and *k* is a background correction factor due to lithospheric effects (with a general values of 1.500). The I_{geo} is divided into seven classes of pollution: $I_{geo} \le 0$ (Class 0 – unpolluted), $0 < I_{geo} < 1$ (Class 1 – unpolluted to moderately polluted), $1 < I_{geo} < 2$ (Class 2 – moderately polluted), $2 < I_{geo} < 3$ (Class 3 – moderately to strongly polluted), $3 < I_{geo} < 4$ (Class 4 – strongly polluted), $4 < I_{geo} < 5$ (Class 5 – strongly to extremely polluted), $I_{geo} \ge 5$ (Class 6 – extremely polluted).

The Potential Ecological Risk Index (PERI) is a consequence of toxicity– response factors and illustrates the sensitivity of various biological communities to metals [8]. It was introduced by Håkanson [4], and it is defined by the following Equation (5):

$$\mathrm{PERI} = \sum_{i=1}^{n} E_r^i = \sum_{i=1}^{n} T_r^i \times C_f^i = \sum_{i=1}^{n} T_r^i \times \frac{C_0^i}{C_n^i} \quad (5)$$

where: PERI is the sum of all risk indices for metals in sediments, E_r^i is the monomial potential ecological risk index, T_r^i is the toxic-response factor for a given substance, C_f^i is the pollution factor, C_0^i is the concentration of metal in sediments, and C_n^i is the background value for a specific metal. T_r^i for Hg, Cr, Cu, Zn, Pb, As and Cd have the next values: 40, 2, 5, 1, 5, 10 and 30, respectively. In order to characterize E_r^i, 5 categories of ecological risk levels were used: $E_r^i < 40$ (low potential ecological risk), $40 \le E_r^i < 80$ (moderate potential ecological risk), $80 \le E_r^i < 160$ (considerable potential ecological risk), $160 \le E_r^i < 320$ (high potential ecological risk) and $320 \le E_r^i$ (very high ecological risk) [9]. Håkanson classification [4] was considered regarding PERI: PERI < 50 (none), 50 ≤ PERI < 120 (low ecological risk), 120 ≤ PERI < 240 (moderate ecological risk), 240 ≤ PERI < 400 (considerable risk), PERI ≥ 600 (very high ecological risk).

A variation of the overall degree of contamination is the modified degree of contamination (mCd) proposed by Abrahim and Parker [10]:

$$mC_d = \frac{\sum_{i=1}^{9} C_f^i}{9} \quad (6)$$

where: C_f^i is the contamination factor obtained for each substance. Classification of sediments based on this modified index is as follows: 0 < mCd < 1.500 (null to a very low degree of contamination), 1.500 ≤ mCd < 2 (low degree of contamination), 2 ≤ mCd < 4 (moderate degree of contamination), 4 ≤ mCd < 8 (high degree of contamination), 8 ≤ mCd < 16 (very high degree of contamination), 16 ≤ mCd < 32 (extremely high degree of contamination), mCd ≥ 32 (ultra-high degree of contamination).

The Metal Pollution Index (MPI) was developed by Usero et al. [11] and is used to show the degree of heavy metal pollution:

$$\mathrm{MPI} = (C_{Mn} \times C_{Zn} \times C_{Cu} \times C_{Pb} \times C_{Sn} \times C_{Cd} \times C_{V} \times C_{As} \times C_{Se})^{1/9} \quad (7)$$

where: C is the concentration of the specific heavy metal expressed in $\mu g\ g^{-1}$ dry weight.

The Pollution Load Index (PLI) proposed by Tomlinson et al. [12] was used to compare the pollution status of different sediment samples and to evaluate the mutual pollution effects of different metals such as Hg, Cd, Pb, As, Cr, Cu and Zn. This index is expressed as follows:

$$\mathrm{PLI} = (C_f^1 \times C_f^2 \ldots .\ C_f^n)^{1/n} \quad (8)$$

where: C_f^n is the contamination factor obtained by dividing each metal's concentration by its corresponding background values. There are two types of PLI, according to Chakravarty and Patgiri [13] and Seshan et al. [14]: PLI < 1 (unpolluted) and PLI > 1 (polluted).

Sediment Quality Guidelines (SQGs) developed by U.S. National Oceanic and Atmospheric Administration (NOAA) and Canadian guidelines for freshwater ecosystems [15] were used to evaluate ecotoxicology and to predict the adverse biological effects caused by chemicals in sediments: the effect range low (ERL), effect range median (ERM),

threshold effect level (TEL) and probable effect level (PEL) values. NOOA guidelines consist of a threshold effect concentration (TEC) below which adverse effects are not expected to occur and a probable effect concentration (PEC) above which adverse effects are expected to occur more often, classifying sediments as rarely (<ERL), occasionally (≥ERL and <ERM) or frequently (>ERM) associated with adverse biological effects [15,16]. Long and MacDonald [17] developed a procedure based on the application of mean PEL quotients (m-PEL-Q) to account for the additive toxicity effects of mixtures of chemicals:

$$\text{m-PEL-Q}=\frac{\sum_{i=1}^{n} C_o/PEL_i}{n} \tag{9}$$

where: C_0 is the concentration of metal in sediments, PEL is the probable effect level of pollutant i and n is the number of pollutants included in the calculation. A specific ranking of sites of potential concern has been proposed based on m-PEL-Q values: m-PEL-Q < 0.100 (lowest priority), 0.110 < m-PEL-Q < 1.510 (medium-low priority), 1.510 < m-PEL-Q < 2.300 (medium-high priority) and m-PEL-Q > 2.300 (high priority).

The mean ERM quotient (m-ERM-Q) is also used as a beneficial tool to distinguish and prioritize the areas of potential hazards in terms of the quality of sediments [18–20]:

$$\text{m-ERM-Q}=\frac{\sum_{i=1}^{n} C_o/ERM_i}{n} \tag{10}$$

where: C_0 is the concentration of metal in sediments, ERM is the effects range-medium of pollutant i and n is the number of pollutants. Several classes of toxicity probability for the biota were defined by Long and MacDonald [17] based on m-ERM-Q: m-ERM-Q < 0.100 (9% probability of toxicity), 0.110 < m-ERM-Q < 0.500 (21% probability of toxicity), 0.510 < m-ERM-Q < 1.500 (49% probability of toxicity) and m-ERM-Q > 1.510 (76% probability of toxicity).

Furthermore, Fairey et al. [21] proposed the implementation of sediment quality guideline quotients (SQGQs) to estimate how many of the thresholds were exceeded on average:

$$\text{SQGQ}=\sum_{i=1}^{n}\left(\frac{\overline{C_0^i}}{MPL_i}+\ldots+\frac{\overline{C_n^i}}{MPL_i}\right)/n \tag{11}$$

where: $\overline{C_0^i}$ is the average concentration of pollutant i in sediments, MPL_i is the maximum permissible limit for pollutant i in sediments from Romanian Legislation [22], i represents pollutants such as Cd, Cu, Ni, Pb, Zn, and n is the number of pollutants. The classification of SQGQs by Abessa et al. [23] includes 4 types of environmental risk: 0 < SQGQ < 0.100 (no risk for benthic organisms), 0.100 ≤ SQGQ < 0.250 (discrete risk), 0.250 ≤ SQGQ < 1 (environment under risk of impact) and SQGQ ≥ 1 (strong risk of impact).

Toxic Units (TUs) also represent useful tools for the normalization of the general toxicity of different heavy metals [24]. They allow the comparison of relative effects induced by the metals and can be defined by the following Equation:

$$TUs_i=\frac{C_o}{PEL_i} \tag{12}$$

where: C_0 is the metal concentration in sediments, and PEL is the probable effect level of pollutant i.

The potential acute toxicity ($\sum TUs$) can be assessed based on the values of TUs and can be calculated as follows [25]:

$$\sum TUs=\sum_{i=1}^{n} TUs_i=\sum_{i=1}^{n}\frac{C_o}{PEL_i} \tag{13}$$

where: C_0 is the concentration of metal in sediments, PEL is the probable effect level of pollutant *i* and *n* is the number of pollutants.

The individual Toxic Risk Index (TRI_i) was also developed to assess the ecotoxicity of a system based on TEL and PEL effects [26]:

$$TRI_i = \sqrt{\frac{\left(\frac{C_0}{TEL}\right)^2 + \left(\frac{C_0}{PEL}\right)^2}{2}} \quad (14)$$

The integrated toxic risk index (TRI) summarizes the total toxicity of several heavy metals in a system by following the next Equation [27]:

$$TRI = \sum_{i=1}^{n} TRI_i = \sum_{i=1}^{n} \sqrt{\frac{\left(\frac{C_0}{TEL}\right)^2 + \left(\frac{C_0}{PEL}\right)^2}{2}} \quad (15)$$

where: C_0 is the concentration of metal in sediments, TEL is the threshold effect level, PEL is the probable effect level of pollutant *i* and n is the number of pollutants. The classification of pollution levels based on TRI values follows the next sequence: TRI ≤ 5 (no toxic risk), 5 < TRI ≤ 10 (low toxic risk), 10 < TRI ≤ 15 (moderate toxic risk), 15 < TRI ≤ 20 (considerable toxic risk), TRI > 20 (very high toxic risk) [26].

The following Equations were used to estimate the heavy metals exposure doses [28]:

$$ADD_{ingestion}\ (mg/kg/day) = \frac{Cw \times IR \times CF \times EF \times ED}{BW \times AT} \quad (16)$$

$$ADD_{dermal}\ (mg/kg/day) = \frac{Cw \times SA \times AF \times ABS \times CF \times EF \times ED}{BW \times AT} \quad (17)$$

$$LADD_{ingestion}\ (mg/kg/day) = \frac{Cw \times IR \times CF \times EF \times ED}{BW \times LT} \quad (18)$$

$$LADD_{dermal}\ (mg/kg/day) = \frac{Cw \times SA \times AF \times ABS \times CF \times EF \times ED}{BW \times LT} \quad (19)$$

$$HQ = \frac{ADD}{RfD} \quad (20)$$

$$HI = \sum HQ = \sum \frac{ADD}{RfD} \quad (21)$$

$$LCR = LADD \times SF \quad (22)$$

$$CNR = HI_{ingestion} + HI_{dermal} \quad (23)$$

$$CCR = LCR_{ingestion} + LCR_{dermal} \quad (24)$$

where: ADD [mg/kg/day] represents the average daily dose from water ingestion/dermal contact, LADD [mg/kg/day] is the lifetime average daily dose from water ingestion/dermal contact, Cw [mg/kg] is the concentration of a particular metal, IR [mg/day] is the sediment ingestion rate (100 mg/day for an adult and 100 mg/day for a child), CF [kg/mg] is the conversion factor (10^{-6} kg/mg), EF [days/year] is the exposure frequency (122 days for an adult and 90 days for a child), ED [years] is the exposure duration (30 years for an adult and 7 years for a child), SA [cm^2] is the exposed skin area (11,153.1 cm^2 for an adult and 4664.6 cm^2 for a child), AF [mg/cm^2] is the sediment adherence factor (0.07 mg/cm^2 for an adult and 0.200 mg/cm^2 for a child), ABS [dimensionless] is the bioavailability factor, BW [kg] is the body weight (is considered 70 kg for an adult and 30 kg for a child), AT [days] is the average time (AT = ED x 365 days/year), LT [days] is the lifetime (years converted to days; usually 70 years x 365 days), HQ [dimensionless] is the hazard quotient, RfD [mg/kg/day] is the chronic reference dose for a particular chemical, HI [dimensionless] is the total exposure hazard index (risk for non-carcinogenic effects), LCR [dimensionless]

is the lifetime risk for carcinogenic effects (dermal or ingestion cancer risk), SF [mg/kg/day] is the cancer slope factor, CNR [dimensionless] is the cumulative non-carcinogenic risk and CCR [dimensionless] is the cumulative carcinogenic risk for both dermal and ingestion scenarios.

The RfD and SF values were obtained from the USEPA-Integrated Risk Information System (IRIS) database. USEPA health regulations assume that if HI <1, then the chemical exposure is less than the benchmark, and it is unlikely to have a potential non-carcinogenic effect, while if HI > 1, there is a chance that non-carcinogenic risks may occur. On the other hand, if LCR < 10^{-6}, the lifetime cancer risk is less than 1 in a million and is typically considered negligible, if 10^{-6} < LCR < 10^{-4} there is a potential carcinogenic risk, whereas if LCR > 10^{-4} the lifetime cancer risk is greater than 1 in ten thousand and screening analysis should be involved due to the high potential carcinogenic risk [29].

S.B. Results of The Metal Pollution Indices in Lacu Sărat Sediments

Zn and Ni show a slight increase in 1946 and 1968 (54 mg/kg and 32 mg/kg, respectively), and between 1993 and 2008 (54 mg/kg and 28 mg/kg), with a higher peak at around 1978 (72 mg/kg and 40 mg/kg, respectively). The lowest values for Zn and Ni were identified in 1988 (42 mg/kg and 26 mg/kg, respectively). Cr registered a significant decrease from 75 mg/kg in 1918 to 55 mg/kg in 1930, followed by small fluctuations until 1978, when it reached a maximum concentration of almost 90 mg/kg. Hg presents a quite different tendency, increasing from 0.010 mg/kg in 1918 to 0.020 mg/kg between 1933 and 1953, 0.030 mg/kg in 1980 and 0.050 mg/kg in 2008. On the other hand, As decreased from 8.500 mg/kg in 1930 to 3 mg/kg in 1963, followed by an increased amount of 5 mg/kg in 1996. The content of Cd reveals two significant peaks of 0.300 mg/kg around 1978 and 2008. High concentrations of Pb were recorded in 1946 (13 mg/kg), 1970 (14 mg/kg), 1978 and 2008 (16 mg/kg), whereas in the case of Mn, the highest values occurred in 1946 (1100 mg/kg), 1968 and 1996 (875 mg/kg). It is noticeable that Co, Al and Fe exhibit important peaks in 1946 (11 mg/kg for Co, 5 mg/kg for Al and 2.800 mg/kg for Fe, respectively), 1970 (11 mg/kg for Co, 5.500 mg/kg for Al and 3 mg/kg for Fe, respectively), 1978 (13 mg/kg for Co, 6.500 mg/kg for Al and 3.600 mg/kg for Fe, respectively) and in 1996 (10 mg/kg for Co, 4.500 mg/kg for Al and 2.600 mg/kg for Fe, respectively).

Table S1. Maximum permissible limits of heavy metals according to various legislations.

Maximum Permissible Limits (mg/kg)	US EPA*			Canada** (CSQG) (mg/kg)	Romania*** (Order 161/2006) (mg/kg)
	Non-Polluted	Moderately Polluted	Heavily Polluted		
**Pb**	< 40	40–60	> 60	35	85
**Cd**	-	-	> 6	0.600	0.800
**Zn**	< 90	90–200	> 200	123	150
**Cu**	< 25	25–50	> 50	35.700	40
**Ni**	< 20	20–50	> 50	-	35
**As**	< 3	3–8	> 8	5.900	29
**Cr**	< 25	25–75	> 75	37.300	100
**Fe**	< 17,000	17,000–25,000	> 25,000	-	-
**Mn**	< 300	300–500	> 500	-	-
**Hg**	< 1	1	> 1	0.170	0.300

*US Environmental Protection Agency (USEPA) 2010 Guidance on Evaluating Sediment Contaminant Results. [30]. US Environmental Protection Agency (EPA) 2007. Sediment Toxicity Identification Evaluation (TIE). Phases I, II, and III Guidance Document [31]
**Canadian Council of Ministers of the Environment (CCME) 2001 – Canadian Environmental Quality Guidelines (CEQG). Canadian sediment quality guidelines for the protection of aquatic life [32].
***Order No 161/2006 for the approval of norms concerning the classification of surface water quality to determine the ecological status of water bodies, Romanian Ministry of Environment and Water, Official Monitor of Romania no. 511 of 13/06/2006 [22].

The heavy metal concentrations registered in the analyzed sediment samples from Lacu Sărat Lake do not exceed the maximum permissible limits provided by Romanian legislation, except for Ni (between 1977 and 1981). Fe, Cd, Pb, Zn and Hg concentrations are lower than the sediment quality standards established by environmental regulatory authorities in Romania, Canada and The United States of America. Sediment samples were moderately polluted with Cu (in the periods of 1964–1969 and 1977–1981) and Ni (between 1918 and 2007), according to US EPA regulations [30]. The investigated samples were also moderately polluted with Cr (in the periods of 1931–1974 and 1988–2014), Mn (between 2008 and 2014) and As (in the periods of 1945–1960 and 1969–2007) and heavily polluted with Cr (in the periods of 1918–1930 and 1977–1981), Mn (1918 and 2007) and As (between 1918 and 1931), as compared to the US EPA standards. Moreover, according to the Canadian sediment quality guidelines [32], the samples were polluted with Cr (between 1918 and 2014) and As (between 1918 and 1931). There were no regulations established for the content of Al and Co in sediments.

Table S2. Values of Cd, mCd, MPI and PLI for investigated heavy metals.

Indices	Cd	mCd	MPI	PLI
Lacu Sărat Lake sediments	9.090	0.820	13.320	0.060

The results reveal the fact that the superficial sediments from Lacu Sărat Lake have been slightly polluted with heavy metals. Thus, these metals present low levels of the contamination factor, except for Cd (2.000), Cr (1.170) and As (1.870), which induce moderate contamination.

Cr values in sediment samples from Lacu Sărat Lake were higher than TEL and TEC in all cases (except for the superficial sample) but lower than PEL and PEC. Cr concentrations were higher than ERL, but lower than ERM at 15 cm depth, the sediments being occasionally associated with adverse biological effects. When it comes to Ni, the concentrations in the investigated sediments are lower than TEL, PEC and PEL values in all cases (except between 12 and 15 cm depth) but higher than TEC (except for the first layer of sediment). Moreover, Ni concentrations exceed ERL values at 6 cm depth, between 12 and 15 cm, between 21 and 24 cm and at 33 cm, but they are lower than ERM, enhancing the fact that the sediments can occasionally represent a risk for the aquatic ecosystems.

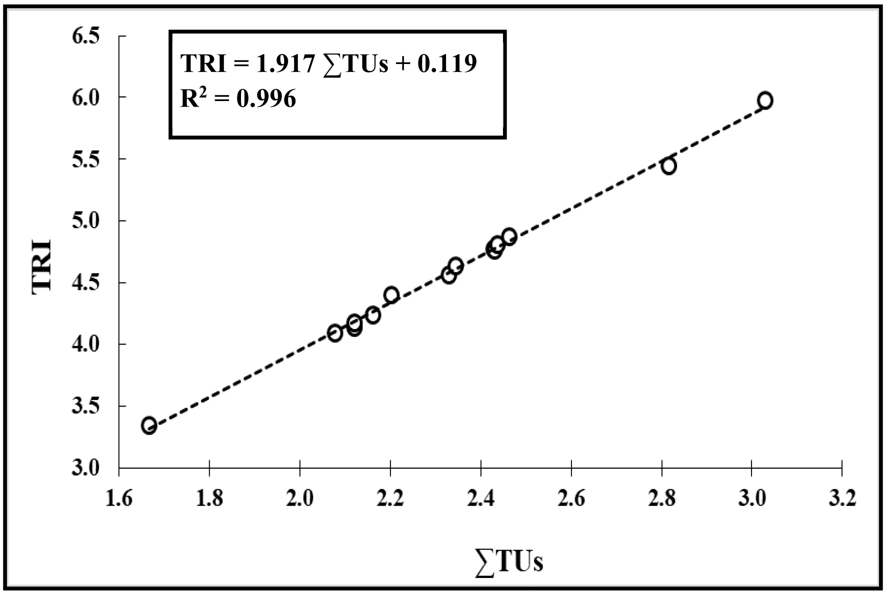

Figure S1. Correlation between TRI and ∑Tus.

The mean values of the individual toxic risk index (TRI) of the investigated metals varied between 0.044 and 1.826, following the same sequence as for the toxic units and indicating a high potential risk for Ni, Cr and As and a low one for Hg and Cd. The temporal fluctuations of TRI enhance significant input to the total risk index for Cr and Ni between 1964 and 1981, for As between 1918 and 1931, and a moderate one for Pb, Zn, Cd and Hg between 2007 and 2014.

Table S3. Exposure doses to heavy metals from sediments considering the accidental ingestion or dermal contact scenarios

Exposure Dose	Recep-tor	Heavy Metal											
		**Pb**	**Cd**	**As**	**Cu**	**Zn**	**Cr**	**Al**	**Ni**	**Mn**	**Hg**	**Fe**	**Co**
**ADD_{dermal} (mg/kg/day)**	**Adult**	5.370 10^{-5}	9.320 10^{-10}	3.800 10^{-7}	6.300 10^{-5}	1.900 10^{-4}	2.160 10^{-6}	1.110 10^{-5}	3.600 10^{-6}	7.510 10^{-5}	1.490 10^{-7}	6.440 10^{-6}	2.500 10^{-5}
	**Children**	1.100 10^{-4}	1.920 10^{-9}	7.820 10^{-7}	1.300 10^{-4}	3.910 10^{-4}	4.430 10^{-6}	2.280 10^{-5}	7.410 10^{-6}	1.540 10^{-4}	3.070 10^{-7}	1.320 10^{-5}	5.140 10^{-5}
**$LADD_{dermal}$ (mg/kg/day)**	**Adult**	2.300 10^{-5}	3.990 10^{-10}	1.630 10^{-7}	2.700 10^{-5}	8.150 10^{-5}	9.240 10^{-7}	4.750 10^{-6}	1.540 10^{-6}	3.220 10^{-5}	6.390 10^{-8}	2.760 10^{-6}	1.070 10^{-5}
	**Children**	1.100 10^{-5}	1.920 10^{-10}	7.820 10^{-8}	1.300 10^{-5}	3.910 10^{-5}	4.430 10^{-7}	2.280 10^{-6}	7.410 10^{-7}	1.540 10^{-5}	3.070 10^{-8}	1.320 10^{-6}	5.140 10^{-6}
**RfD_{dermal}**		**3.500 10^{-3}**	**1.000 10^{-9}**	**2.700 10^{-7}**	**3.700 10^{-2}**	**3.000 10^{-1}**	**5.070 10^{-7}**	**1.000**	**3.200 10^{-5}**	**2.240 10^{-4}**	**1.600 10^{-4}**	**7.000 10^{-1}**	**3.000 10^{-4}**
**$ADD_{ingestion}$ (mg/kg/day)**	**Adult**	6.880 10^{-6}	1.190 10^{-7}	1.620 10^{-6}	8.070 10^{-6}	2.440 10^{-5}	2.120 10^{-5}	1.420 10^{-6}	1.150 10^{-5}	2.400 10^{-4}	1.910 10^{-8}	8.250 10^{-7}	3.200 10^{-6}
	**Children**	2.370 10^{-5}	4.110 10^{-7}	5.590 10^{-6}	2.780 10^{-5}	8.380 10^{-5}	7.310 10^{-5}	4.880 10^{-6}	3.970 10^{-5}	8.280 10^{-4}	6.580 10^{-8}	2.840 10^{-6}	1.100 10^{-5}
**$LADD_{ingestion}$ (mg/kg/day)**	**Adult**	2.950 10^{-6}	5.120 10^{-8}	6.960 10^{-7}	3.460 10^{-6}	1.040 10^{-5}	9.100 10^{-6}	6.080 10^{-7}	4.940 10^{-6}	1.030 10^{-4}	8.190 10^{-9}	3.530 10^{-7}	1.370 10^{-6}
	**Children**	2.370 10^{-6}	4.110 10−8	5.590 10^{-7}	2.780 10^{-6}	8.380 10^{-6}	7.310 10^{-6}	4.880 10^{-7}	3.970 10^{-6}	8.280 10^{-5}	6.580 10^{-9}	2.840 10^{-7}	1.100 10^{-6}
**RfD_{oral}**		**3.500 10^{-3}**	**1.000 10^{-3}**	**3.000 10^{-4}**	**3.700 10^{-2}**	**3.000 10^{-1}**	**3.000 10^{-3}**	**1.000**	**2.000 10^{-2}**	**1.400 10^{-1}**	**1.600 10^{-4}**	**7.000 10^{-1}**	**3.000 10^{-4}**

The light grey colour indicates exceeding the reference doses.

S.C. Results of Statistical Analysis Applied on The Heavy Metals Content in Lacu Sărat Sediments

Table S4. Descriptive statistics of investigated heavy metals.

Descriptive Statistics					
**Metals**	***N***	**Min**	**Max**	**Mean**	**SD**
**Mn**	14	387.3	1084.4	763.5	179.4
**Cr**	14	41.060	88.98	63.07	12.060
**Co**	14	5.100	13.000	9.990	1.940
**Cu**	14	15.500	31.700	21.810	4.510
**Pb**	14	10.000	16.700	12.640	2.220
**Zn**	14	41.000	73.00	50.79	8.270
**Ni**	14	19.800	40.800	30.690	5.470
**As**	14	2.800	8.300	4.580	1.720
**Cd**	14	0.200	0.300	0.210	0.040
**Hg**	12	0.010	0.050	0.020	0.010
**Al**	14	2.510	6.510	4.730	0.950
**Fe**	14	1.460	3.620	2.540	0.520

Table S5. Pearson correlation matrix of the investigated metals from Lacu Sărat Lake sediments.

Pearson’s Correlations

	Mn	Cr	Co	Cu	Pb	Zn	Ni	As	Cd	Hg	Al	Fe
**Mn**	**1**											
**Cr**	**0.253**	1										
**Co**	0.428	0.782	1									
**Cu**	0.122	0.738	0.867	1								
**Pb**	−0.211	0.267	0.385	0.606	1							
**Zn**	−0.050	0.611	0.618	0.776	0.895	1						
**Ni**	0.154	0.729	0.937	0.916	0.599	0.742	1					
**As**	0.718	0.141	0.041	−0.264	−0.242	−0.103	−0.147	1				
**Cd**	−0.248	0.189	0.145	0.300	0.756	0.728	0.272	−0.155	1			
**Hg**	−0.530	−0.493	−0.405	−0.301	0.554	0.234	−0.180	−0.222	0.505	1		
**Al**	0.372	0.857	0.965	0.851	0.276	0.575	0.884	0.017	0.108	−0.505	1	
**Fe**	0.276	0.832	0.968	0.940	0.440	0.688	0.944	−0.098	0.215	−0.399	0.975	1

Two-tailed test of significance is used.
*. Correlation is significant at the 0.05 level.

The degree of correlation among heavy metals can indicate the mobility or sources of sediments (higher coefficients show one source, while lower ones reveal more sources) [33,34]. The results suggest that high positive correlations ($r > 0.800$) were observed between Cr – Al, Cr – Fe, Cu – Co, Ni–Co, Al – Co, Fe – Co, Ni – Cu, Al – Cu, Fe – Cu, Zn – Pb, Ni – Al, Ni – Fe and Al – Fe. Moderate positive correlations ($0.700 < |r| < 0.800$) were noticed for As – Mn, Co – Cr, Cu – Cr, Ni – Cr, Zn – Cu, Cd – Pb, Zn – Ni and Zn – Cd. Other positive correlations ($0.400 < |r| < 0.700$) were found between Co – Mn, Zn – Cr, Zn – Co, Pb – Cu, Al – Zn, Fe – Zn and Cd – Hg. Negative correlations occurred for Pb - Mn, Zn - Mn, Cd - Mn, Hg – Mn, Cr – Hg, Co – Hg, As – Cu, Hg – Cu, As – Pb, As – Zn, As – Ni, Hg – Ni, As – Cd, As – Hg, As – Fe, Hg – Al and Hg – Fe.

Table S6. Principal component analysis for selected heavy metals in lake sediments from Lacu Sărat Lake.

Metals	Rotated Component Matrix		
	PC1	PC2	PC3
Fe	0.992	-	-
Al	0.979	-	-
Co	0.966	-	-
Cu	0.961	-	-
Ni	0.935	-	-
Cr	0.827	-	0.376
Cd	-	0.892	-
Pb	0.400	0.860	-
Hg	−0.474	0.762	-
Zn	0.643	0.739	-
As	-	-	0.971
Mn	0.327	-	0.771
**Initial Eigenvalues**	**6.285**	**2.850**	**1.869**
**% of Variance**	**52.37**	**23.753**	**15.573**
**Cumulative %**	**52.37**	**76.13**	**91.70**

References

1. Loska, K.; Cebula, J.; Pelczar, J.; Wiechuła, D.; Kwapuliński, J. Use of Enrichment, and Contamination Factors Together with Geoaccumulation Indexes to Evaluate the Content of Cd, Cu, and Ni in the Rybnik Water Reservoir in Poland. *Water, Air, and Soil Pollution 1997 93:1* **1997**, *93*, 347–365, doi:10.1023/A:1022121615949.
2. Meena, N.K.; Prakasam, M.; Bhushan, R.; Sarkar, S.; Diwate, P.; Banerji, U. Last-Five-Decade Heavy Metal Pollution Records from the Rewalsar Lake, Himachal Pradesh, India. *Environ Earth Sci* **2017**, *76*, 1–10, doi:10.1007/S12665-016-6303-0/FIGURES/3.
3. Duodu, G.O.; Goonetilleke, A.; Ayoko, G.A. Comparison of Pollution Indices for the Assessment of Heavy Metal in Brisbane River Sediment. *Environmental Pollution* **2016**, *219*, 1077–1091, doi:10.1016/J.ENVPOL.2016.09.008.
4. Hakanson, L. An Ecological Risk Index for Aquatic Pollution Control.a Sedimentological Approach. *Water Res* **1980**, *14*, 975–1001, doi:10.1016/0043-1354(80)90143-8.
5. Islam, M.S.; Hossain, M.B.; Matin, A.; Islam Sarker, M.S. Assessment of Heavy Metal Pollution, Distribution and Source Apportionment in the Sediment from Feni River Estuary, Bangladesh. *Chemosphere* **2018**, *202*, 25–32, doi:10.1016/J.CHEMOSPHERE.2018.03.077.
6. Goher, M.E.; Farhat, H.I.; Abdo, M.H.; Salem, S.G. Metal Pollution Assessment in the Surface Sediment of Lake Nasser, Egypt. *The Egyptian Journal of Aquatic Research* **2014**, *40*, 213–224, doi:10.1016/J.EJAR.2014.09.004.
7. Muller G. Schwermetalle in Den Sedimenten Des Rheins: Veränderungen Seit 1971. *Umschau* **1979**, *79*, 778–783.
8. Yi, Y.; Yang, Z.; Zhang, S. Ecological Risk Assessment of Heavy Metals in Sediment and Human Health Risk Assessment of Heavy Metals in Fishes in the Middle and Lower Reaches of the Yangtze River Basin. *Environmental Pollution* **2011**, *159*, 2575–2585, doi:10.1016/J.ENVPOL.2011.06.011.
9. Hilton, J.; Davison, W.; Ochsenbein, U. A Mathematical Model for Analysis of Sediment Core Data: Implications for Enrichment Factor Calculations and Trace-Metal Transport Mechanisms. *Chem Geol* **1985**, *48*, 281–291, doi:10.1016/0009-2541(85)90053-1.
10. Abrahim, G.M.S.; Parker, R.J. Assessment of Heavy Metal Enrichment Factors and the Degree of Contamination in Marine Sediments from Tamaki Estuary, Auckland, New Zealand. *Environmental Monitoring and Assessment 2007 136:1* **2007**, *136*, 227–238, doi:10.1007/S10661-007-9678-2.
11. Usero, J.; González-Regalado, E.; Gracia, I. Trace Metals in the Bivalve Mollusc Chamelea Gallina from the Atlantic Coast of Southern Spain. *Mar Pollut Bull* **1996**, *32*, 305–310, doi:10.1016/0025-326X(95)00209-6.
12. Tomlinson, D.L.; Wilson, J.G.; Harris, C.R.; Jeffrey, D.W. Problems in the Assessment of Heavy-Metal Levels in Estuaries and the Formation of a Pollution Index. *Helgoländer Meeresuntersuchungen 1980 33:1* **1980**, *33*, 566–575, doi:10.1007/BF02414780.
13. Chakravarty, M.; Patgiri, A.D. Metal Pollution Assessment in Sediments of the Dikrong River, N.E. India. *Journal of Human Ecology* **2017**, *27*, 63–67, doi:10.1080/09709274.2009.11906193.
14. Seshan, B.R.R.; Natesan, U.; Deepthi, K. Geochemical and Statistical Approach for Evaluation of Heavy Metal Pollution in Core Sediments in Southeast Coast of India. *International Journal of Environmental Science & Technology 2010 7:2* **2010**, *7*, 291–306, doi:10.1007/BF03326139.
15. MacDonald, D.D.; Ingersoll, C.G.; Berger, T.A. Development and Evaluation of Consensus-Based Sediment Quality Guidelines for Freshwater Ecosystems. *Archives of Environmental Contamination and Toxicology 2000 39:1* **2000**, *39*, 20–31, doi:10.1007/S002440010075.
16. Bakan, G.; Özkoç, H.B. An Ecological Risk Assessment of the Impact of Heavy Metals in Surface Sediments on Biota from the Mid-Black Sea Coast of Turkey. *http://dx.doi.org/10.1080/00207230601125069* **2007**, *64*, 45–57, doi:10.1080/00207230601125069.
17. Long, E.R.; MacDonald, D.D. Recommended Uses of Empirically Derived, Sediment Quality Guidelines for Marine and Estuarine Ecosystems. *http://dx.doi.org/10.1080/10807039891284956* **2010**, *4*, 1019–1039, doi:10.1080/10807039891284956.
18. Long, E.R.; Macdonald, D.D.; Smith, S.L.; Calder, F.D. Incidence of Adverse Biological Effects within Ranges of Chemical Concentrations in Marine and Estuarine Sediments. *Environmental Management 1995 19:1* **1995**, *19*, 81–97, doi:10.1007/BF02472006.

19. McCready, S.; Birch, G.F.; Long, E.R. Metallic and Organic Contaminants in Sediments of Sydney Harbour, Australia and Vicinity — A Chemical Dataset for Evaluating Sediment Quality Guidelines. *Environ Int* **2006**, *32*, 455–465, doi:10.1016/J.ENVINT.2005.10.006.
20. Violintzis, C.; Arditsoglou, A.; Voutsa, D. Elemental Composition of Suspended Particulate Matter and Sediments in the Coastal Environment of Thermaikos Bay, Greece: Delineating the Impact of Inland Waters and Wastewaters. *J Hazard Mater* **2009**, *166*, 1250–1260, doi:10.1016/J.JHAZMAT.2008.12.046.
21. Fairey, R.; Long, E.R.; Roberts, C.A.; Anderson, B.S.; Phillips, B.M.; Hunt, J.W.; Puckett, H.R.; Wilson, C.J. An Evaluation of Methods for Calculating Mean Sediment Quality Guideline Quotients as Indicators of Contamination and Acute Toxicity to Amphipods by Chemical Mixtures. *Environ Toxicol Chem* **2001**, *20*, 2276–2286, doi:10.1002/ETC.5620201021.
22. Romanian Ministry of Environment and Water *Order No 161/2006 for the Approval of Norms Concerning the Classification of Surface Water Quality to Determine the Ecological Status of Water Bodies*; 2006;
23. Abessa DMS, C.R.S.E.R.B.Z.L.P.Y.G.M.B.M.H.M.S.J.M.P. Integrative Ecotoxicological Assessment of a Complex Tropical Estuarine System. In *Marine Pollution: New Research*; Hofer Tobias N., Ed.; Nova Science Publishers Inc. : New York, 2008; pp. 1–72.
24. Iqbal, J.; Shah, M.H.; Shaheen, N. Distribution, Source Identification and Risk Assessment of Selected Metals in Sediments from Freshwater Lake. *International Journal of Sediment Research* **2015**, *30*, 241–249, doi:10.1016/J.IJSRC.2012.12.001.
25. Pedersen, F.; Bjørnestad, E.; Andersen, H.V.; Kjølholt, J.; Poll, C. Characterization of Sediments from Copenhagen Harbour by Use of Biotests. *Water Science and Technology* **1998**, *37*, 233–240, doi:10.2166/WST.1998.0757.
26. Zhang, Z.; Juying, L.; Mamat, Z. Sources Identification and Pollution Evaluation of Heavy Metals in the Surface Sediments of Bortala River, Northwest China. *Ecotoxicol Environ Saf* **2016**, *126*, 94–101, doi:10.1016/J.ECOENV.2015.12.025.
27. Ranjbar Jafarabadi, A.; Riyahi Bakhtiyari, A.; Shadmehri Toosi, A.; Jadot, C. Spatial Distribution, Ecological and Health Risk Assessment of Heavy Metals in Marine Surface Sediments and Coastal Seawaters of Fringing Coral Reefs of the Persian Gulf, Iran. *Chemosphere* **2017**, *185*, 1090–1111, doi:10.1016/J.CHEMOSPHERE.2017.07.110.
28. Newman, M.C.; Diamond, G.L.; Menzie, C.; Moya, J.; Nriagu, J. *Issue Paper On Metal Exposure Assessment*; Washington DC, 2004;
29. Albering, H.J.; Rila, J.P.; Moonen, E.J.C.; Hoogewerff, J.A.; Kleinjans, J.C.S. Human Health Risk Assessment in Relation to Environmental Pollution of Two Artificial Freshwater Lakes in The Netherlands. *Environ Health Perspect* **1999**, *107*, 27–35, doi:10.1289/EHP.9910727.
30. Division of Surface Water (USEPA) *Guidance on Evaluating Sediment Contaminant Results*; Washington DC, 2010;
31. Ho K.T., B.R.M., M.D.R., N.-K.T.J., H.J. *Sediment Toxicity Identification Evaluation (TIE). Phases I, II, and III Guidance Document*; Washington DC, 2007;
32. Canadian Council of Ministers of the Environment (CCME) *Canadian Environmental Quality Guidelines (CEQG). Canadian Sediment Quality Guidelines for the Protection of Aquatic Life: Introduction. Updated*; Winnipeg, 2001;
33. Pradit, S.; Wattayakorn, G.; Angsupanich, S.; Baeyens, W.; Leermakers, M. Distribution of Trace Elements in Sediments and Biota of Songkhla Lake, Southern Thailand. *Water Air Soil Pollut* **2010**, *206*, 155–174, doi:10.1007/S11270-009-0093-X/TABLES/7.
34. Tang, W.; Ao, L.; Zhang, H.; Shan, B. Accumulation and Risk of Heavy Metals in Relation to Agricultural Intensification in the River Sediments of Agricultural Regions. *Environ Earth Sci* **2014**, *71*, 3945–3951, doi:10.1007/S12665-013-2779-Z/FIGURES/5.
